# On Diabetic and Nephritic Neuralgias

**Published:** 1882-06

**Authors:** 


### ORIGINAL TRANSLATIONS AND ABSTRACTS.

### ON DIABETIC AND NEPHRITIC NEURALGIAS.

Prof. Berger, in a lecture before the *Breslau Medical Section,* spoke of neuralgia as a symptom of glycosuria and chronic nephritis. Berger does not allude to undefined rheumatoid or neuralgiform pains, but real neuralgia, accompanied by all the nosographic characters of this affection. The neuralgia may be the only marked symptom, while the morbid condition which underlies it may give rise to no characteristic symptoms, and so give no opportunity for diagnosis. In this lies the diagnostic importance of this form of symptomatic neuralgia. Although several well observed cases of diabetic neuralgia have been reported by Worms, and more recently by Drasche, there are no similar observations on record concerning the neuralgia accompanying nephritis. Bartels, in speaking of the early headaches in nephritis, refers to rare cases of neuralgic attacks in the course of other nerve-tracks, but seems to have remained in doubt whether to bring the neuralgia in connection with the kidney affection.

Berger has observed twenty-one cases of neuralgia dependent upon kidney affections, twelve of which accompanied diabetes, and nine nephritis. In all of these cases the neuralgia led to the diagnosis of the kidney affection. The sciatic plexus is oftenest involved (twelve cases out of the twenty-one were sciatica.)

The important question whether there are any peculiarities about these neuralgias is answered by Berger in the affirmative, and he calls attention to the following points of peculiarity : 1. The spontaneousness of the nerve affection ; the closest examination disclosed no cause for the neuralgia. 2. The limitation of the neuralgia to certain terminal branches of the sciatic. 3. The tendency to symmetrical distribution. 4. The extraordinary severity and long duration of the neuralgic attacks, resembling the spinal neuralgia occurring in lesions of the posterior columns. 5. The marked prominence of vaso-motor manifestations in the areas of the affected nerves. 6. The resistance to the ordinary methods of treatment. 7. The improvement following proper treatment of the kidney affection (regulation of the diet and tonics).

The percentage of sugar in the urine in the several cases varied from 1.9 to 6.7. In general, it may be said that the neuralgia varies in intensity as the percentage of sugar. The neuralgias in the nephritic cases all occurred in patients in the advanced stages of the disease. All of these manifested the circulatory disturbances characterizing chronic Bright's disease, and six of the nine cases had (at the time of writing) died. Berger opposes the suggestion of Drasche that the neuralgia in the diabetic cases is due to the action of the sugar on the peripheral nerves. He believes it to have a centric origin. The nephritic neuralgia he considers as a symptom of uremia. Although the neuralgia in Bright's disease may be believed to be merely a coincidence, the small number of observations not permitting a positive expression of opinion, yet it would be well to keep in mind that an obstinate neuralgia may not only be dependent upon diabetes, but on chronic nephritis.

